# Dynamic sampling for SAXSTT: towards real-time measurement adaptation

**DOI:** 10.1107/S1600577526005308

**Published:** 2026-06-19

**Authors:** Sici Wang, Leonard C. Nielsen, Marianne Liebi

**Affiliations:** a Center for Photon Science Paul Scherrer Institut (PSI) Villigen Switzerland; b Institute of Materials École Polytechnique Fédérale de Lausanne (EPFL) Lausanne Switzerland; c Chalmers e-Commons, Department of Physics Chalmers University of Technology Gothenburg Sweden; Australian Synchrotron, Australia

**Keywords:** small-angle X-ray scattering, tensor tomography, spherical sampling, progressive sampling

## Abstract

Small-angle X-ray scattering tensor tomography (SAXSTT) is a powerful technique for non-destructive 3D characterization of nanoscale structures by reconstructing *q*-resolved reciprocal space maps. Yet this approach requires extensive raster scanning from multiple directions, leading to long acquisition times that challenge synchrotron-based experiments. To overcome this, we propose a dynamic sampling strategy combined with an online error feedback mechanism. This strategy ensures efficient real-time acquisition by adaptively terminating measurements based on evolving reconstruction quality. By applying this strategy to experimental datasets, we demonstrate up to a sevenfold reduction in the required number of projections while maintaining reconstruction quality, with the achievable reduction remaining strongly sample-dependent. Furthermore, we conduct quantitative evaluations using orientation alignment and normalized cross-correlation, confirming the robustness of the presented strategy. Compatible with existing beamline systems, this strategy offers a practical solution for time-efficient and adaptive SAXSTT acquisition.

## Abstract 2

A dynamic sampling strategy is presented to reduce the measurement time required for SAXS tensor tomography while maintaining reconstruction quality.

## 1. Introduction

The development of high-brilliance synchrotron X-ray sources has greatly advanced non-destructive characterization in a wide range of materials (Baruchel *et al.*, 2008 ▸; Willmott, 2019 ▸; Lifshin, 2008 ▸; Bertsch & Hunter, 2001 ▸). Among the available techniques, small-angle X-ray scattering (SAXS) is particularly effective for probing nanoscale structures by analyzing scattering intensity profiles (Pilz *et al.*, 1979 ▸; Koch *et al.*, 2003 ▸). Its combination with scanning has further enabled broad applications in biology and materials science (Fratzl *et al.*, 1997 ▸; Rinnerthaler *et al.*, 1999 ▸), by significantly extending the accessible region of interest. Although scanning SAXS is excellent at mapping out the structures of thin samples, it faces limitations with thicker samples, as the scattering is averaged over the full sample thickness. To overcome this limitation, small-angle X-ray scattering computed tomography (SAXS-CT) was developed by combining SAXS with tomographic acquisition, enabling 3D reconstruction from multi-angle scans around one rotation axis from 0° to 180° (Feldkamp *et al.*, 2009 ▸; Schroer *et al.*, 2006 ▸).

However, SAXS-CT relies on approximate rotational invariance. When this assumption is not met, anisotropic signals may be inaccurately reconstructed, leading to reconstruction artifacts and possible misinterpretation (Schroer *et al.*, 2006 ▸; Stribeck *et al.*, 2006 ▸).

Small-angle X-ray scattering tensor tomography (SAXSTT) addresses the limitation by introducing an additional tilt axis into the conventional SAXS-CT setup. This enables the reconstruction of *q*-resolved reciprocal space maps (RSMs) that fully describe 3D orientation distribution of nano­structures, making the method highly effective for anisotropic samples (Schaff *et al.*, 2015 ▸; Liebi *et al.*, 2015 ▸). As a result, SAXSTT has been increasingly adopted in synchrotron facilities for a wide range of scientific research (Appel *et al.*, 2026 ▸; Georgiadis *et al.*, 2021 ▸; Grünewald *et al.*, 2023 ▸; Barreto *et al.*, 2024 ▸), and recent advances have further enhanced its practicality (Gao *et al.*, 2019 ▸; Nielsen *et al.*, 2023 ▸). In terms of algorithmic improvements, the open source package *Mumott* has largely improved reconstruction time from hours to minutes without compromising accuracy (Nielsen *et al.*, 2025 ▸). Additionally, higher-flux X-ray sources and advances in detector technology have shortened acquisition times by an order of magnitude (Appel *et al.*, 2026 ▸).

**Figure 1:**
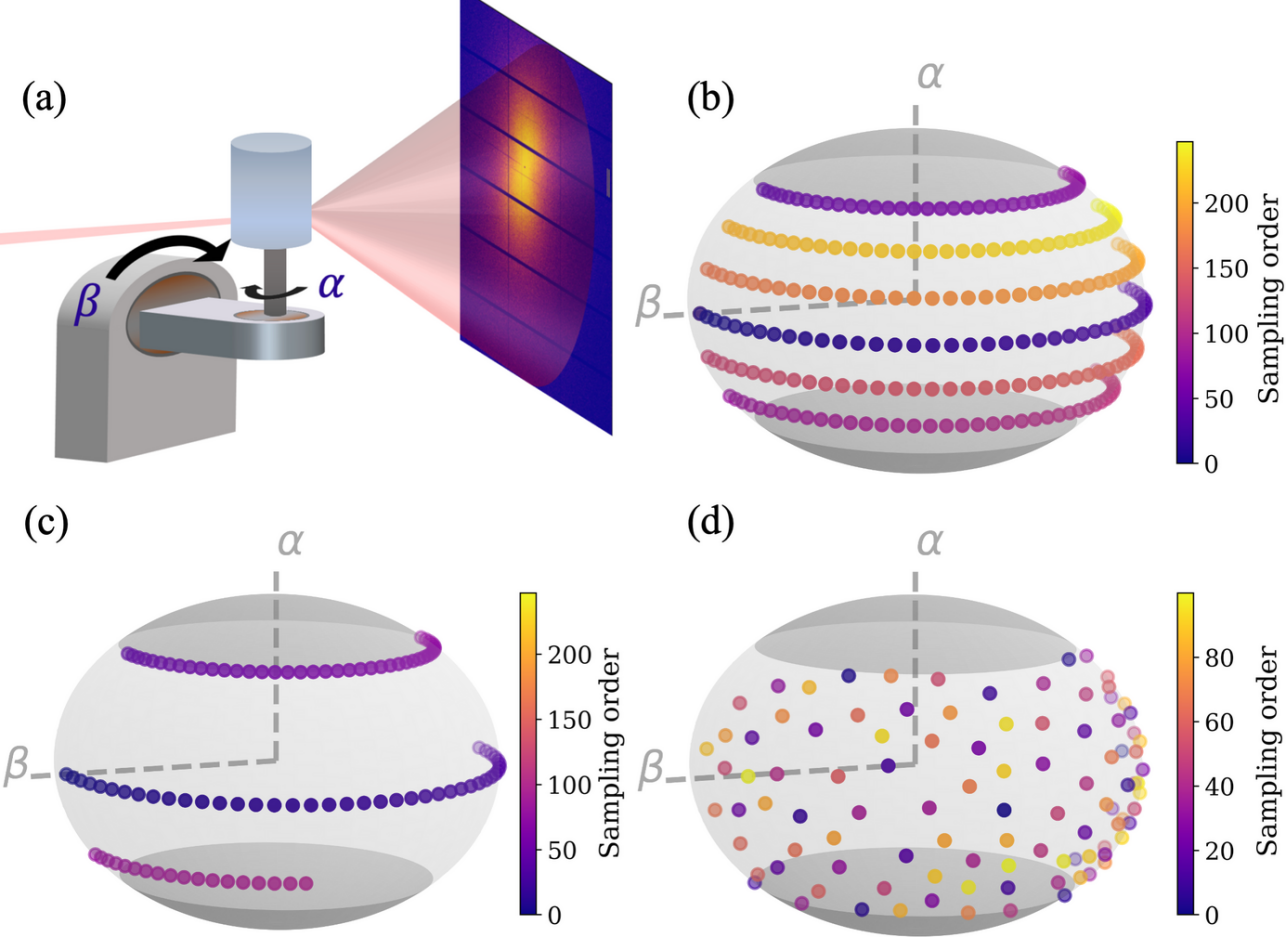
(*a*) Schematic of a typical SAXSTT setup with two rotational axes. (*b*) Equivalent projection sphere showing a heuristic quasi-uniform distribution of 240 experimental projections, with approximately 40 rotations at six distinct tilt angles, and blocked projection directions (dark gray) due to geometric constraints. (*c*) Sequential acquisition of the first 100 projections results in clustered sampling along specific latitudes on the projection sphere. (*d*) Sequential acquisition of 100 projections generated by our max–min sampling strategy distribute quasi-uniformly.

Despite these advances, SAXSTT remains limited by relatively long acquisition times, as the addition of a tilt axis expands the sampling domain from a half-circle to a half-sphere, as illustrated in Fig. 1 ▸(*b*). This substantially increases the sampling requirement to several hundred projections. So far, this projection number has been predetermined according to an empirical rule, namely to measure six times the number needed for scalar tomography, as introduced in the first implementation (Liebi *et al.*, 2015 ▸). However, the required projection number depends on the complexity of the sample in both real and reciprocal space, and is therefore difficult to predict before a measurement. In addition to the pre­determined projection number, the projection sequence is also executed in a fixed order (Liebi *et al.*, 2015 ▸). This reflects a typical SAXSTT setup, where dual-axis goniometers naturally favor completing one tilt series before moving to the next. Thus, it is necessary to wait until all projections are acquired, since any interruption may cause uneven angular sampling and leave the dataset insufficient for reconstruction. As a result, such a heuristic acquisition scheme may lead to inefficient use of beam time when fewer projections would be sufficient. It has been shown that, for samples that are sparse in real space, such as trabecular bone, manual removal of most already acquired projections in an offline analysis can still preserve the reconstruction quality (Liebi *et al.*, 2018 ▸). Moreover, long exposure to high-flux X-rays increases the risk of radiation damage to sensitive samples, further limiting the method’s applicability (Holton, 2009 ▸; Grünewald *et al.*, 2024 ▸).

To address these limitations, we propose a novel SAXSTT acquisition framework that integrates a dynamic uniform sampling strategy with real-time error feedback. This framework enables projections to be added incrementally based on the previous projection directions, allowing the measurement to be terminated once sufficient data have been collected. In this way, the information obtained with each measurement can be maximized, while angular uniformity and structural fidelity are preserved.

Beyond SAXSTT, we expect this strategy to extend to wide-angle X-ray scattering tensor tomography (Frewein *et al.*, 2024 ▸) and directional-dark field tensor tomography (Kim *et al.*, 2021 ▸), where similar challenges have been reported.

## 2. Methods

A typical SAXSTT setup is illustrated in Fig. 1 ▸(*a*), where the sample is illuminated by a focused X-ray beam and rotated along two axes, denoted 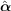 and 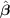. Although the X-ray beam itself remains fixed, the dual-axis rotation of the sample effectively generates a set of projections across a spherical angular space. This allows us to conceptualize the projection process on a unit sphere, as shown in Fig. 1 ▸(*b*), where each dot represents a unique projection direction. The dark gray areas indicate missing wedge regions, which are commonly encountered in tomography acquisitions because the beam is obstructed by the sample holder (Nielsen *et al.*, 2024 ▸).

In practice, the choice of projection directions in SAXSTT experiments is constrained by several factors, such as the sample size and the chosen beam size to match the desired resolution. According to the Crowther criterion (Klug & Crowther, 1972 ▸), ideal sampling at zero tilt would require several hundred projections, approximately 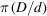, for a mm-scale sample *D* illuminated by a micrometre-scale beam *d*. When more tilts are included, the required projection number will increase beyond a thousand, corresponding to hundreds of acquisition hours, making such experiments impractical at synchrotrons. Therefore, the current projection scheme largely follows the empirical rule of thumb from the first implementation (Liebi *et al.*, 2015 ▸), reducing the sampling density suggested by the Crowther criterion and producing six semicircular trajectories distributed across several latitudes, as shown in Fig. 1 ▸(*b*).

In addition to this empirical choice of projection directions, the acquisition order itself can also be constrained by the use of dual-axis goniometers, where changing the tilt axis can induce increased overheads depending on the implementation. As a result, all rotation steps at a given tilt are typically completed before moving to the next tilt.

This rigid order makes the acquisition process highly vulnerable to interruptions or radiation damage effects, potentially resulting in non-uniform angular coverage if the scan is stopped in advance, as shown in Fig. 1 ▸(*c*). Furthermore, due to the absence of reconstruction feedback during data acquisition, the measurement typically proceeds until all pre-determined projections are collected, placing a significant demand on synchrotron beam time (Liebi *et al.*, 2015 ▸).

Here we propose to overcome these limitations through a new projection strategy that enables dynamic uniform sampling guided by an online reconstruction. Instead of a two-stage setup, this strategy takes advantage of advanced motorized goniometers that provide enhanced angular flexibility, enabling continuous and versatile orientation control during acquisition (Appel *et al.*, 2026 ▸). In addition, the reconstruction quality is continuously monitored, thus the projection can be terminated once an ideal error threshold is reached. This approach ensures sufficient sampling while avoiding unnecessary projections, thereby reducing overall measurement time.

### 2.1. Dynamic uniform sampling

Previous studies have proposed stairwise and spiral sampling to speed up tensor tomography by reducing acquisition overhead (Kim *et al.*, 2021 ▸). In addition, Fibonacci sampling provides an effective way to achieve near-uniform spherical coverage by placing points along golden spirals (González, 2010 ▸). However, all of these methods rely on a predefined number of projections and therefore cannot adapt dynamically during acquisition.

Dynamically distributing points in a uniform manner is a common problem across several fields. In physics, simplified *energy minimization* models describe how electrons on a sphere repel each other to form well spaced configurations (Erber & Hockney, 1991 ▸). In mathematics, *farthest point sampling* iteratively selects the point farthest from the current set to maintain even spacing (Eldar *et al.*, 1997 ▸). In biology, *Tammes’s problem* was introduced to explain pollen grain patterns, aiming to find a globally optimal arrangement of points on a sphere (Tammes, 1930 ▸).

Inspired by these studies, here we introduce a strategy *max–min sampling*, in which each new sampling point is chosen to maximize its minimal angular distance to all previously selected points. Formally, the next point 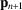 is selected according to the criterion
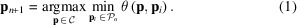
Here, 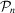 = 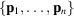 denotes the set of *n* sampling points selected previously, and 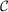 is the candidate pool of potential samplings. The set 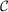 can be defined to ensure that only physically feasible directions are considered during sampling, *e.g.* taking missing-wedge limitations into account. The angular distance between two unit vectors 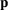 and 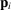 is defined as 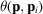 = 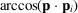. In addition, due to tomographic symmetry, we apply a wrapping condition that restricts the effective angular distance to a maximum of 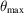 = 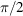. Points beyond this threshold are reflected across the center of the sphere and treated as symmetrically equivalent. The wrapped angular distance θ_wrapped_ between two unit vectors 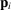 and 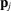 is defined as
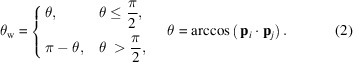
This treatment ensures that the calculated distance never exceeds 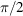, which effectively folds the sphere and allows all sampling points to be considered within a hemisphere region.

By defining 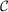, selecting 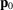, and iteratively applying equations (1) and (2), each subsequent sampling point is placed in the sparsest region of the sphere, ensuring uniform angular coverage regardless of the total number of projections. The first 100 points generated by this strategy are shown in Fig. 1 ▸(*d*), where the color transition from purple to yellow indicates the sampling order.

**Figure 2:**
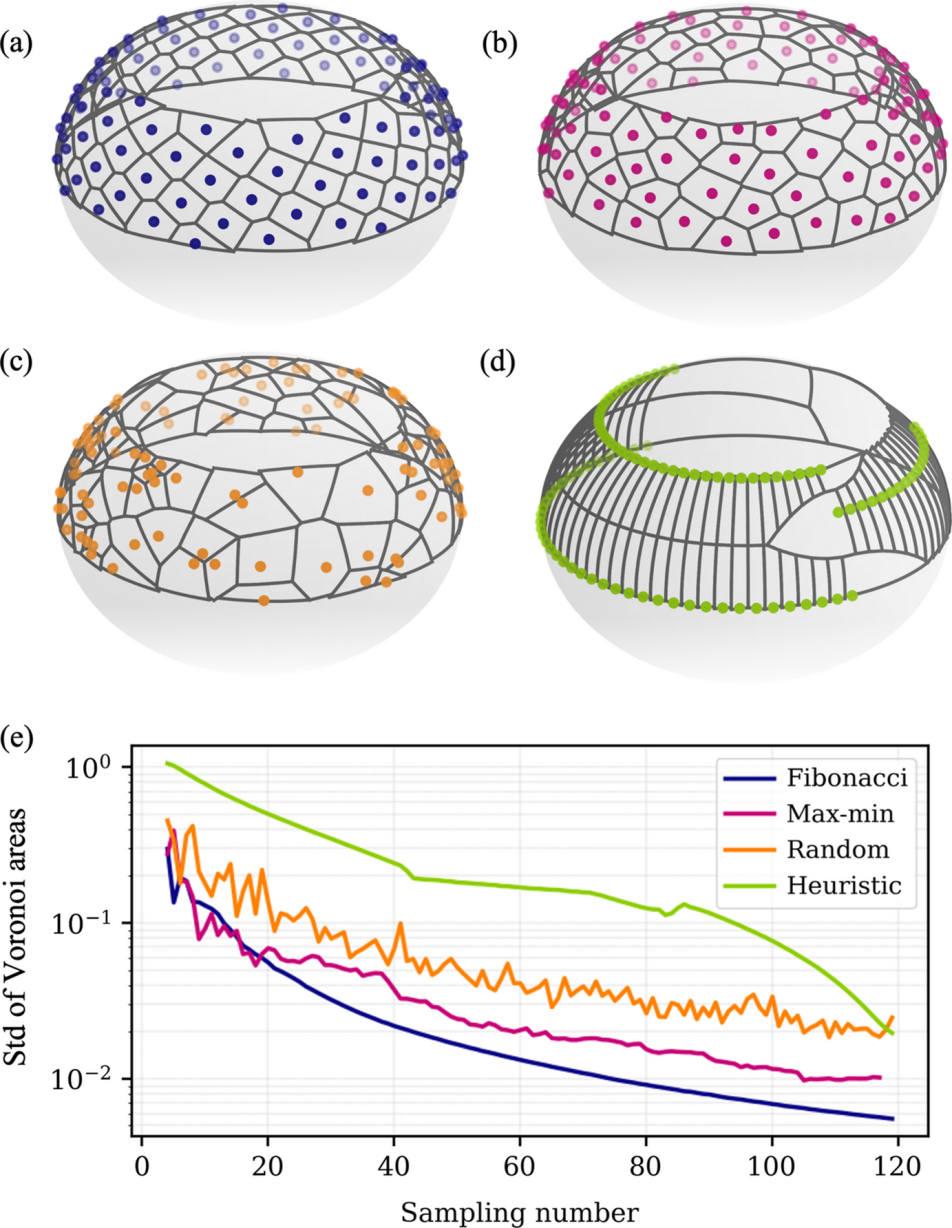
Distributions of 100 sampling points and corresponding spherical Voronoi diagrams for (*a*) Fibonacci, (*b*) our proposed max–min, (*c*) random and (*d*) 240-projection heuristic preset, with the missing-wedge region considered. (*e*) Comparison of the standard deviation in Voronoi region areas across four strategies, as the sampling number increases from 4 to 120.

To evaluate the angular uniformity of different sampling strategies, we employed a quantitative analysis based on Voronoi diagrams (Aurenhammer & Klein, 1996 ▸). A Voronoi diagram divides the space into regions, each corresponding to the area closest to an individual sampling point. When the points are uniformly distributed, these regions are expected to be similar in both size and shape. Consequently, the standard deviation of their region sizes provides a quantitative metric to evaluate the sampling uniformity. In this case, we applied this analysis to four sampling strategies: Fibonacci sampling, our proposed max–min sampling, random sampling and the experimental heuristic sampling designed for 240 projections. Figs. 2 ▸(*a*) to 2(*d*) illustrate the distribution of 100 sampling points on the sphere and their corresponding Voronoi diagrams. The evolution of the standard deviation metric as sampling number increases from 4 to 120 is shown in Fig. 2 ▸(*e*). Note that the projections are illustrated on the upper hemisphere, with α ranging from 0 to 360° and β from 0 to 45°. This configuration is often preferred experimentally because it minimizes collision constraints when only one tilt direction is required. It is equivalent to considering the front hemisphere as in Fig. 1 ▸(*b*).

As expected, Fibonacci sampling consistently yields the lowest standard deviation across all sampling numbers, consistent with its well established uniformity. The heuristic scheme shows the highest level of non-uniformity, with distinct stepwise jumps corresponding to changes between successive tilt angles, clearly visible at sampling numbers 42 and 83. Notably, the heuristic scheme can even be less uniform than random sampling. The purely random acquisition order is included here only as a reference, as it exhibits large fluctuations and is unrealistic for SAXSTT experiments. In contrast, our proposed max–min strategy shows a rapid and steady increase in uniformity with sampling number. Overall, max–min sampling lies between random and Fibonacci sampling under this metric, but remains much closer to the Fibonacci reference, particularly once the sampling number exceeds 20.

### 2.2. Real-time error feedback

Liebi *et al.* previously demonstrated that SAXSTT reconstructions of a trabecular bone sample can be achieved using far fewer projections than the heuristic sampling suggests (Liebi *et al.*, 2018 ▸).

In that study, several reduced projection subsets were generated by downsampling the full dataset of 240 projections at regular angular intervals, corresponding to different projection numbers and sampling schemes. Reconstruction was performed using each subset, and the resulting reconstructed RSMs were forward projected along all 240 original projection directions. The predicted SAXS intensities were then compared with the complete measured dataset, providing a normalized error curve that served as a benchmark for assessing the reconstruction performance of the reduced projection subsets. The comparison showed that a well distributed 44-projection subset could achieve errors comparable with those obtained using the full dataset, and, in some cases, outperform a larger but more clustered 120-projection subset, emphasizing the importance of angular distribution rather than projection number alone. Since these reduced subsets are defined retrospectively after the full dataset has been acquired, we refer to this approach as *static uniform sampling*.

**Figure 3:**
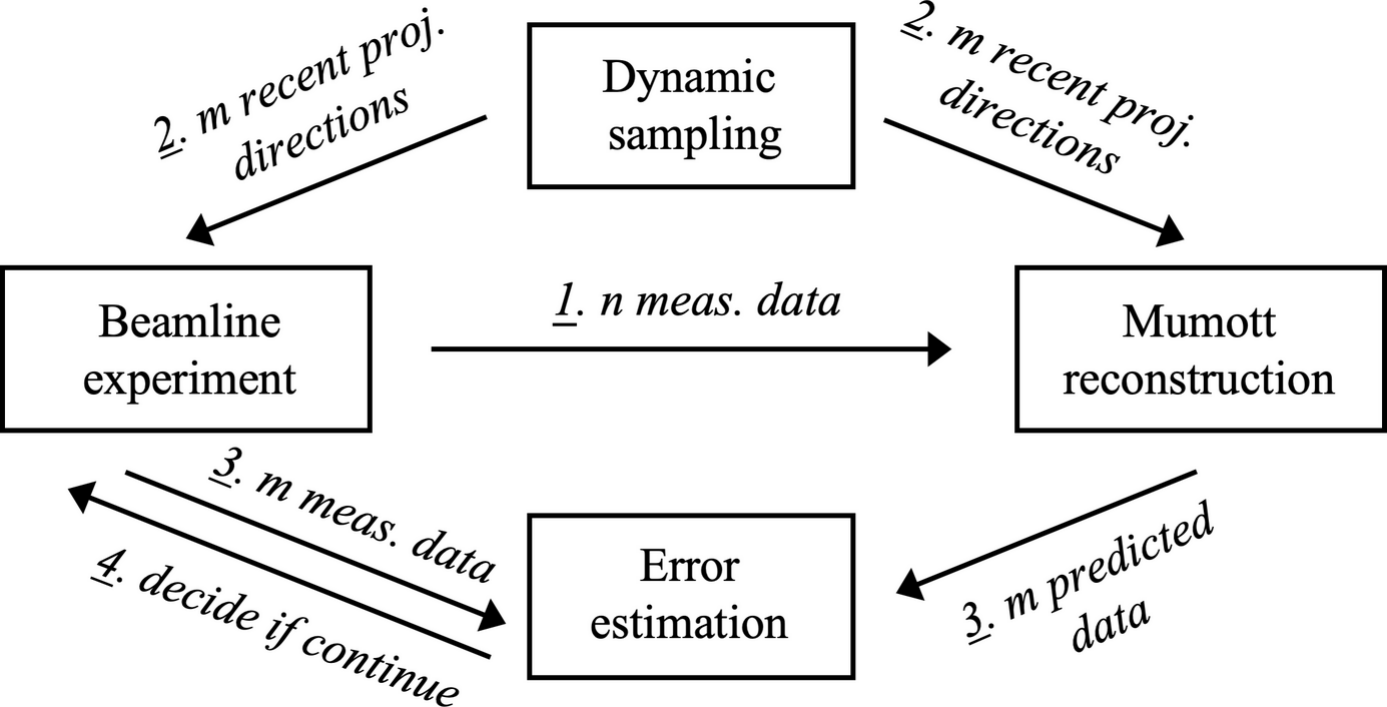
Flow of the dynamic uniform sampling framework with real-time error feedback: (1) (*n* − *m*) projections in *n* measured projections are used for an initial reconstruction; (2) the dynamic sampling strategy determines the most recently *m* projection directions; (3) predicted data for these *m* directions are generated by *Mumott* from initial reconstruction, while the beamline acquires the real measurements; (4) the difference between *m* measured and predicted data determines whether to continue the acquisition.

However, in the dynamic sampling framework presented here, measurements can be terminated at any stage, leaving no fixed reference dataset or benchmark for comparison. To address this, we adopt an alternative error estimation strategy: for *n* acquired projections, 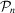 = 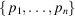, the reconstruction is performed using only the first (*n* − *m*) projections, {*p*_1_,…, *p*_*n*–*m*_}, while the error is evaluated against the *m* most recently acquired measurements, {*p*_*n*–*m*+1_,…, *p*_*n*_}, which are excluded from the reconstruction. This separation between reconstruction and evaluation data prevents artificially low errors caused by including evaluation projections in the reconstruction, analogous to the separation of training and validation data concept in machine learning. Since the error from a single projection may fluctuate due to sample anisotropy and local artifacts, the evaluation error is averaged over *m* > 1 evaluation projections to obtain a more stable metric. As the projections are selected by the max–min sampling strategy, these evaluation projections are distributed far apart in angular space, ensuring that the averaged error is not biased toward a particular region of the reconstruction. The flow of the whole framework is shown in Fig. 3 ▸.

### 2.3. SAXSTT experiments

#### 2.3.1. Human vertebral trabecular bone sample

As described in detail previously (Liebi *et al.*, 2018 ▸), trabecular bone was extracted from the 12th thoracic (T12) vertebra of a 73-year-old male donor obtained from the Department of Anatomy, Histology and Embryology, Innsbruck Medical University, Innsbruck, Austria. The specimen was provided with written donor consent in accordance with Austrian law, and all subsequent procedures complied with Swiss regulations, including the Guideline on Bio-Banking of the Swiss Academy of Medical Sciences (2006) and the Swiss Ordinance 814.912 (2012) on the contained use of organisms. Soft tissue was removed before embedding the vertebra in polymethyl methacrylate (PMMA), and a cylindrical region of approximately 1 mm in diameter was milled from the PMMA block to obtain the sample. Measurements were performed at the cSAXS beamline (X12SA) of the Swiss Light Source, Paul Scherrer Institut, using a monochromatic X-ray energy of 12.4 keV, a beam size of approximately 25 µm × 25 µm, and a step size of 25 µm. A total of 247 projections, each consisting of 55 × 65 scanning points, were recorded with an exposure time of 30 ms per point. The angular spacing was 15° between −30° and 45° in β, and 4.5° between 0° and 180° in α. After each tilt, a measurement at β = 0° was repeated, so the available projections are 240. The total exposure time was 8.3 h, corresponding to 7.2 s per voxel, while the overall measurement duration including motor movements was 20.3 h. The SAXS signal was analyzed over a *q*-range of 0.0379–0.075 nm^−1^, corresponding to real-space distances of approximately 165–85 nm. The data are available at https://zenodo.org/records/10074598.

#### 2.3.2. Carbon-fiber PEEK sample

A cylindrical specimen (approximately 1 mm × 1 mm × 2 mm) was extracted about 7 mm from the edge of a thermoplastic composite plate consisting of carbon fibers (CF) embedded in poly(ether ether ketone) (PEEK). The composite was produced by consolidation of PEEK–CF chips under high temperature and pressure, where the chips were derived from unidirectional CF–PEEK prepregs. Measurements were performed at the cSAXS beamline (X12SA) of the Swiss Light Source, Paul Scherrer Institut, using an X-ray energy of 12.4 keV, a beam and step size of 30 µm, and an exposure time of 40 ms per point. A total of 423 projections, corresponding to 1.76 million scattering patterns, were acquired at eight tilt angles (β = 0°, 10°, 20°, 25°, 30°, 35°, 40°, 50°). For each tilt angle, the effective rotation step size was defined as 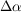 = 5.45°cosβ. The rotation angle α spanned 0°–180° at β = 0° and 0°–360° for all remaining tilt angles. After each tilt, a measurement at α = 0° was repeated to check for potential radiation damage. The data are available at https://doi.org/10.5281/zenodo.17713339.

## 3. Results and discussions

To assess the effectiveness of our framework, we applied it to two existing SAXSTT experimental datasets mentioned above and one simulation data.

### 3.1. Experimental data

We first compared three sampling strategies for selecting 40, 80 and 120 projections from the full dataset of 240 projections on a trabecular bone sample: (1) static uniform sampling (SUS) selects projections at regular angular intervals, mimicking the manually down-sampling method used by Liebi *et al.* (2018 ▸), and serves as a useful benchmark for evaluating alternative strategies; (2) static non-uniform sampling (SNUS) refers to the heuristic SAXSTT procedures, mimicking an interrupted measurement; (3) our proposed dynamic uniform sampling (DUS).

**Figure 4:**
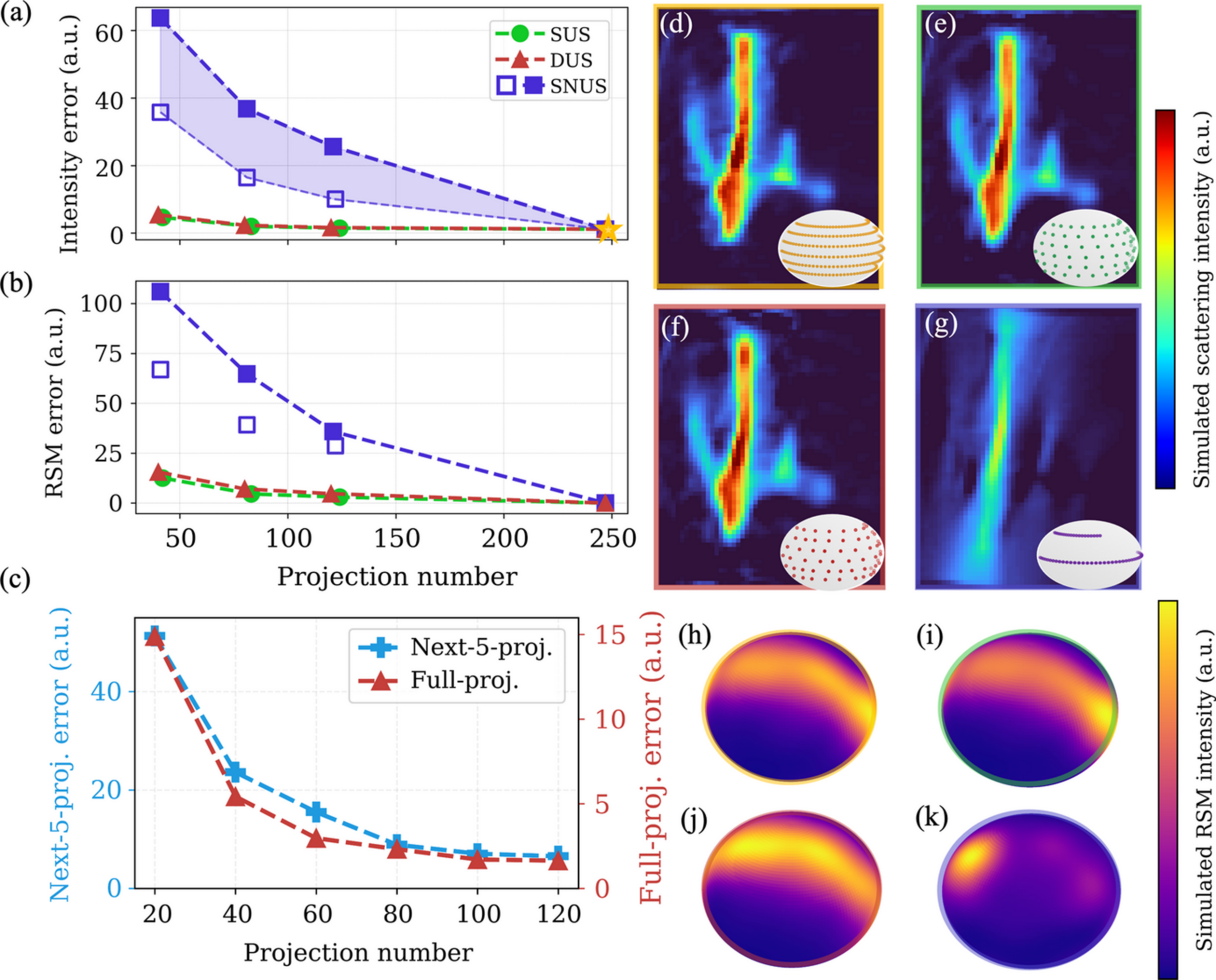
(*a*) Intensity error computed between the predicted and measured data using the full projection matrix. The yellow star indicates that the error is zero when the full dataset is used for reconstruction. (*b*) RSM reconstruction error under the same conditions. (*c*) Comparison of intensity errors between full-projection and next-five-projection. (*d*) Predicted intensity of random direction (83rd) from using the full dataset (after azimuthal binning processing). Predicted intensities from reconstructions using 60 projections by (*e*) SUS, (*f*) DUS and (*g*) SNUS (corresponding to open-square curve), while the insets illustrate the corresponding distributions. Reconstructed RSM in a random single voxel using (*h*) the full dataset, (*i*) 40 SUS, (*j*) 40 DUS and (*k*) 40 SNUS projections (corresponding to the open-square curve).

Reconstructions were performed using *Mumott* (Nielsen *et al.*, 2025 ▸) initially, with no regularization applied. At this stage, regularization terms were intentionally excluded to focus purely on fitting the measured data, rather than improving the reconstruction quality itself. For each sampling strategy, the reconstructed RSMs were then forward-projected using the full projection matrix, ensuring that predicted intensities *I*^pred^ are always compared against all 240 measured projections *I*^meas^, then the residual norm is defined by
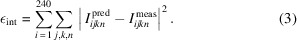
where *i* denotes the projection number, *j* and *k* indicate the raster-scanning position, and *n* denotes the azimuthal bin index. *I*_*ijkn*_ represents the intensity integrated over the selected *q* range within the *n*th azimuthal sector of the annulus. As shown in Fig. 4 ▸(*a*), SUS consistently yields the lowest intensity error across all projection numbers, due to its regular and evenly distributed angular coverage. Violet squares represent SNUS commonly used in current SAXSTT experiments. The filled square corresponds to an extreme case where sampling starts at higher tilt angles (*e.g.* 45°, 30°), resulting in more clustered directions and larger errors when projection numbers are limited. The open square describes a more gentle acquisition path starting near the 0° tilt, with more balanced spacing, though still with significant deviation from the uniform strategy. The shaded region between the violet curves visualizes the uncertainty range of sequential acquisition arising from different starting tilt angles. In contrast, our proposed DUS achieves both low errors and consistent performance across projections. Even with only 40 projections, the error approaches that of SUS and improves steadily with more projections, demonstrating its robustness for dynamic SAXSTT acquisition. These quantitative trends are further supported by visual inspection of the forward-projected predicted intensities obtained from reconstructions based on the full dataset and 60 projections, as shown in Figs. 4 ▸(*d*)–4(*g*). To illustrate this, we examine a randomly selected predicted intensity pattern (corresponding to the 83rd projection direction). The predictions from (*e*) 60 SUS and (*f*) 60 DUS projections closely follow (*d*) the prediction derived from the full-projection reconstruction, whereas (*g*) 60 SNUS projections exhibits a noticeable degradation in quality, consistent with the filled-square markers in the error curve.

Moreover, to evaluate reconstruction quality more directly, we further compared each RSM reconstruction with a reference generated from all 240 projections. Here, regularizers such as Total Variation and Huber Norm are applied to promote spatial smoothness and suppress local artifacts, aiming to best preserve the underlying RSM structure. The RSM in each voxel was represented by a vector of 72 Gaussian kernel coefficients. To compensate for the varying kernel density on the spherical grid, a weighting factor *w*_*i*_ was assigned to each kernel according to its representative area on the sphere. The reconstruction error was then defined as the weighted sum of squared residuals between the reconstructed and reference coefficient vectors, 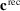 and 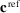, over all voxels *v*, 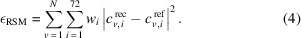
Notably, the error trend in Fig. 4 ▸(*b*) closely follows that of the intensity-based curve in Fig. 4 ▸(*a*). This correspondence is further supported by the voxel-wise RSM reconstruction visualized in Figs. 4 ▸(*h*) to 4(*k*). The RSM reconstructed from (*j*) 40 DUS projections closely resembles that from (*i*) SUS and (*h*) the reference, whereas the result from (*k*) SNUS shows clear degradation. As expected, these qualitative differences align well with the quantitative error trends.

As described in *Methods*[Sec sec2], we also need to compare two error-evaluation methods to support on-the-fly decisions about when to stop. The first is the full-projection error [red triangles in Fig. 4 ▸(*a*)]. The second uses only the next several projections excluded from reconstruction, reflecting dynamic acquisition conditions. In this case, to account for varying intensity scales across projections, each error is normalized by the corresponding measured intensity before averaging, as given by the equation 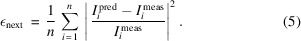
As shown in Fig. 4 ▸(*c*), although the two error curves differ in scale, they exhibit similar overall trends: both decrease rapidly with the projection number up to around 60, after which the improvement slows and the curves begin to plateau.

Comparing all three error curves in Figs. 4 ▸(*a*) to 4(*c*) further reveals that the full-projection intensity error acts as a bridge between the other two: the next-few-projection error, which is available in real time, and the RSM reconstruction error, which more directly reflects structural fidelity but is less accessible due to the time-consuming iterative tuning of the optimal regularization parameters. This correspondence supports the use of the next-few-projection error as a practical indicator of the reconstruction quality, allowing adaptive sampling guided by real-time feedback. By monitoring the rate of error reduction, it becomes possible to identify when additional projections provide only limited improvement, allowing acquisition to be terminated at a suitable point.

**Figure 5:**
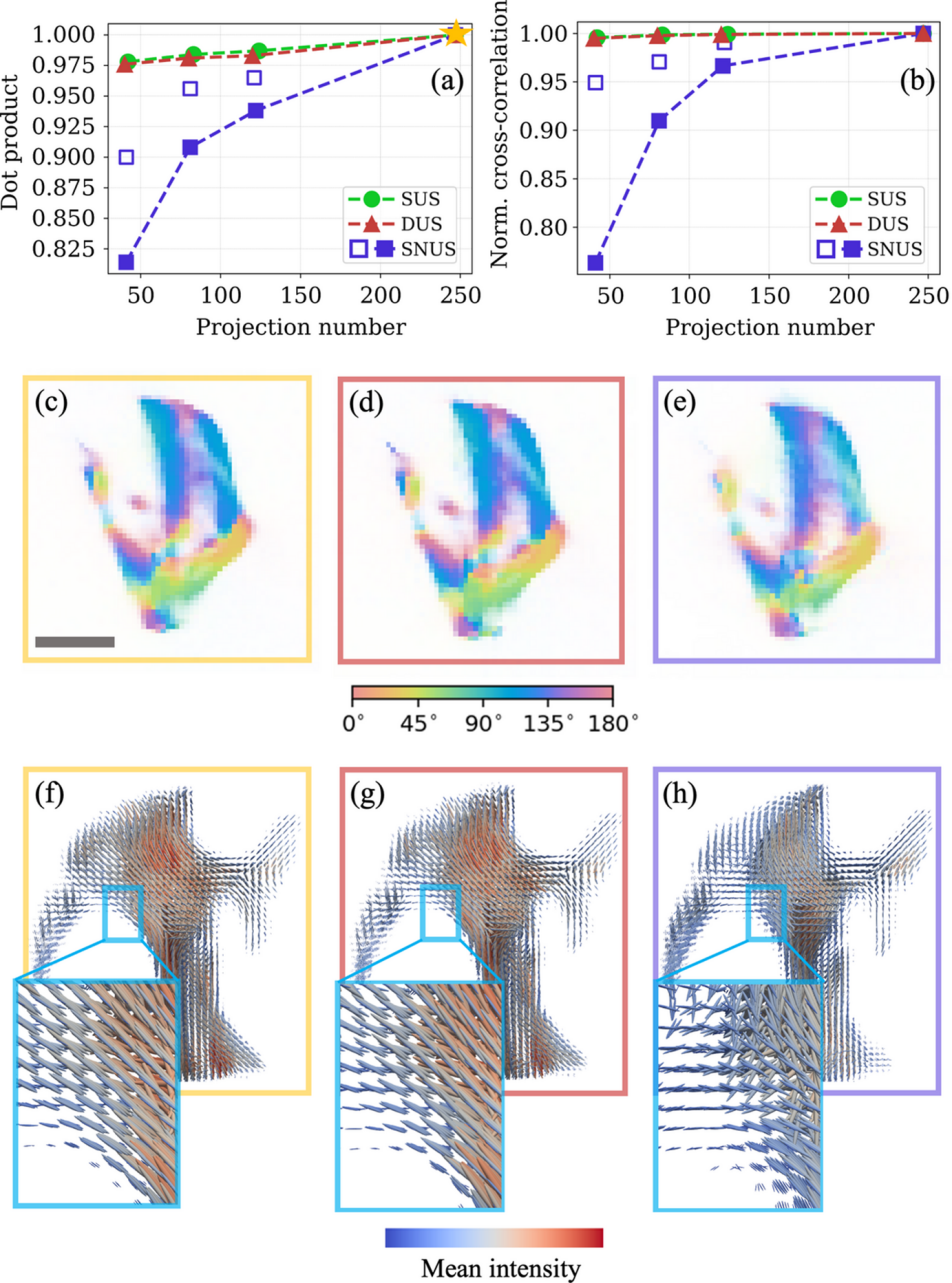
(*a*) Main orientation alignment scores across different sampling strategies and projection numbers. The yellow star indicates a score of 1 obtained using the full dataset for reconstruction. (*b*) Normalized cross-correlation coefficients between reconstructed and reference RSM under the same conditions. Orientation slice map reconstructed from (*c*) the full dataset where color indicating the local direction, (*d*) 40 DUS projections and (*e*) 120 SNUS projections corresponding to the filled-square marker in (*a*) and (*b*) (scale bar represents 500 µm). Glyph render of orientation and mean value in each voxel using (*f*) the full dataset, (*g*) 40 DUS projections and (*h*) 120 SNUS projections [same marker as (*e*)].

In addition to standard error metrics, we further evaluated structural fidelity by other criteria. For orientation alignment, we extracted voxel-wise main orientations, which represent an essential feature quantified by SAXSTT, from the reconstructed RSMs and compared them with the reference within the masked sample region. The alignment score was then defined as the weighted mean absolute dot product (Guizar-Sicairos *et al.*, 2020 ▸),
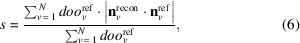
where 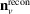 and 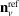 denote the reconstructed and the reference main orientation vectors in voxel *v*, and *N* is the voxel number in the sample mask. The weight *doo* (degree of orientation) increases with anisotropy (0 for isotropic), and the denominator normalizes the weights, ensuring 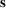

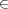
 [0, 1]. Fig. 5 ▸(*a*) compares alignment scores across different sampling strategies and projection counts. Both SUS and DUS maintain high alignment scores (above 0.97) even with only 40 projections, whereas SNUS performs significantly worse throughout. Fig. 5 ▸(*c*) shows the orientation map of a reference volume slice, with color indicating local plane orientation. Although (*e*) 120 SNUS projections were used, the result is less accurate than (*d*) 40 DUS projections, which better matches the reference.

On the other hand, to evaluate global structural similarity, we further computed the normalized cross-correlation coefficient (Lewis, 1995 ▸),
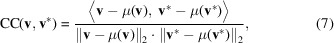
where 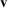 and 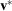 denote the mean value of each RSM in the reconstruction and in the reference dataset, respectively. The notation 〈·, ·〉 denotes the Euclidean inner product, ∥ · ∥_2_ represents the L2 norm, and 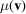, 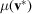 denote the average of 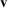 and 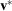 over all voxels.

The cross-correlation coefficient in Fig. 5 ▸(*b*) closely follow the orientation alignment results. Both DUS and SUS yield high similarity to the reference, while SNUS performs noticeably worse. This trend is further supported by the glyph renders in Figs. 5 ▸(*f*) to 5(*h*). Compared with (*h*) 120 SNUS projections, reconstruction from (*g*) only 40 DUS projections shows better agreement with (*f*) the reference. This is evident in several aspects, including the overall sample shape, the color distribution representing the mean value, and the small cylinders indicating the local orientation. These results further confirm the effectiveness of DUS.

Since the projection number required for SAXSTT can be sample-dependent, we also tested the proposed strategy on a second experimental specimen. Unlike the sparse trabecular bone sample, this specimen is a solid cylindrical composite consisting of layered carbon-fiber structures embedded in a PEEK matrix, with each layer exhibiting a distinct orientation. It was measured with 423 projections, and reconstructions were performed using 60 and 150 projections selected using different sampling strategies.

**Figure 6:**
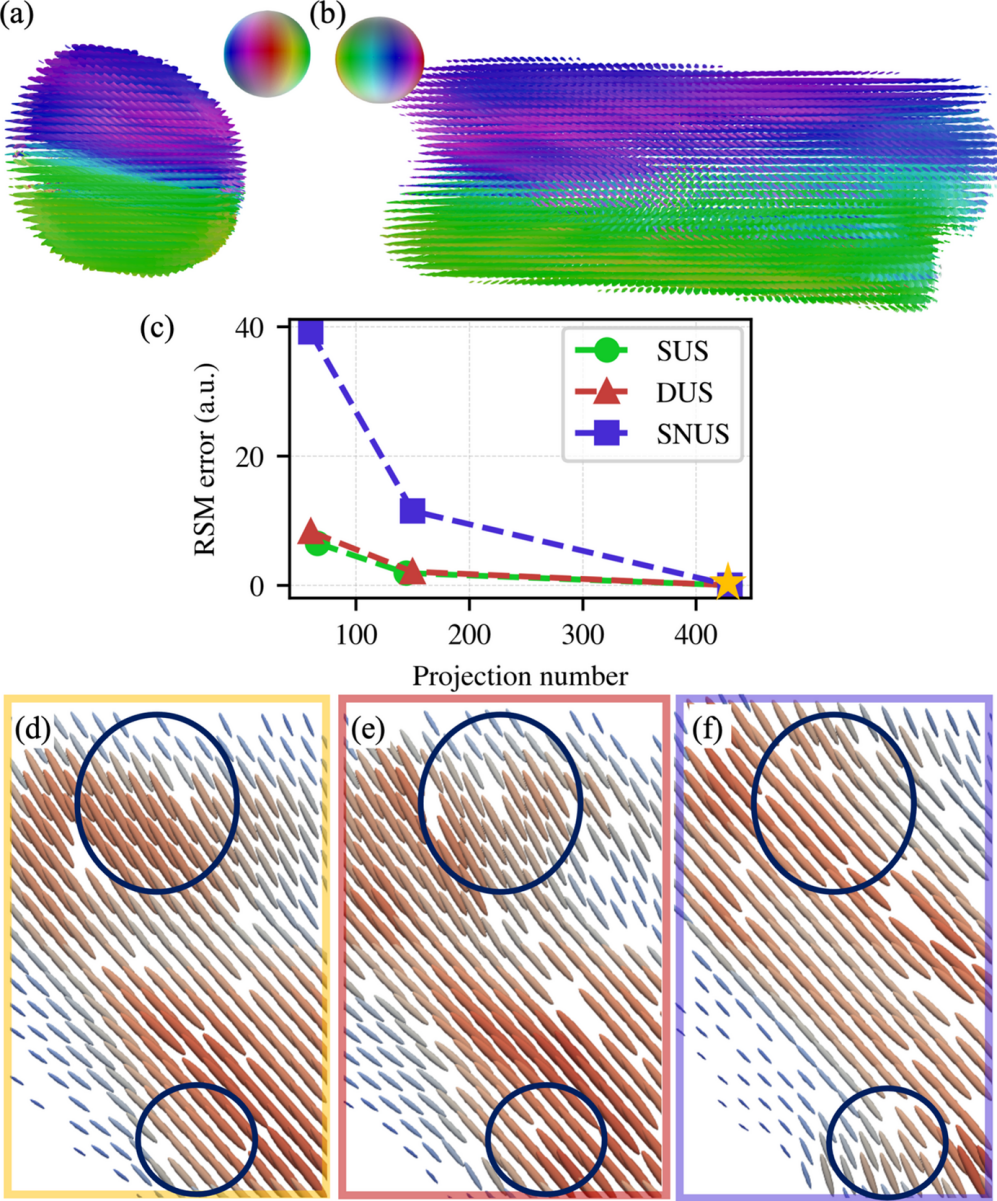
Color-coded spheres indicate two main orientations within the layered carbon-fiber-in-PEEK sample: (*a*) side view and (*b*) front view. (*c*) RSM error curves correspond to different projection numbers and sampling strategies. The yellow star indicates the full-dataset reconstruction. (*d*)–(*f*) One representative reconstructed slice from the purple-pink part, where color indicates the mean value and the cylinder orientation represents the main direction; (*d*) from full 423 projections as reference, (*e*) from 60 DUS projections, in which key orientation features are preserved, and (*f*) from the first 60 SNUS projections, showing more obvious difference.

Figs. 6 ▸(*a*) and 6(*b*) show side and front views of the sample, where the purple–pink and green regions represent two dominant orientations, consistent with the manufacturer’s note of unidirectional alignment within individual layers. In Fig. 6 ▸(*c*), DUS yielded substantially lower RSM reconstruction error than SNUS in agreement with the results on trabecular bone sample. A representative slice taken from one of the two dominant orientation regions is visualized in Figs. 6 ▸(*d*) to 6(*f*). Fig. 6 ▸(*d*) shows the result from the full dataset, while Fig. 6 ▸(*e*) shows the result using only 60 DUS projections. Despite the reduced data, key structural features and dominant orientations are well preserved, indicating that approximately 1/7 of the full dataset is sufficient for reliable reconstruction in this case. In contrast, the reconstruction based on the first 60 heuristic projections fails to capture detailed features and shows inconsistencies with the reference, as shown in Fig. 6 ▸(*f*).

So far, our analysis mainly focus on experimentally acquired data. Although such data are essential for validating the method under realistic conditions, they inherently limit flexibility in point selection, as sampling is restricted to directions that were actually measured, thus equation (1) should be adjusted to
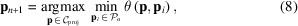
where 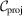, the candidate pool, is restricted in the existing projections. In addition, for the experimental data, the only available ‘reference’ is the reconstruction from the full dataset, whereas in simulations the true ‘ground truth’ is known and can be used for direct comparison.

### 3.2. Simulation sample

Here we conducted a simulation using synthetic data (Nielsen *et al.*, 2023 ▸), available at https://doi.org/10.5281/zenodo.7673985. The simulated sample allows unrestricted selection of projection directions throughout the whole accessible angular space, hereafter referred to as *random* max–min.

The simulation workflow is as follows: (1) a synthetic sample with known structural orientation is generated to serve as ‘ground truth’; (2) a set of projections is applied to simulate measured SAXS data via forward projection with artificial Poisson noise; (3) the simulated data are then reconstructed using the same algorithm as above; (4) reconstruction outputs are compared with the ‘ground truth’ in terms of RSM error, voxel-wise mean intensity, anisotropy level and main orientation.

**Figure 7:**
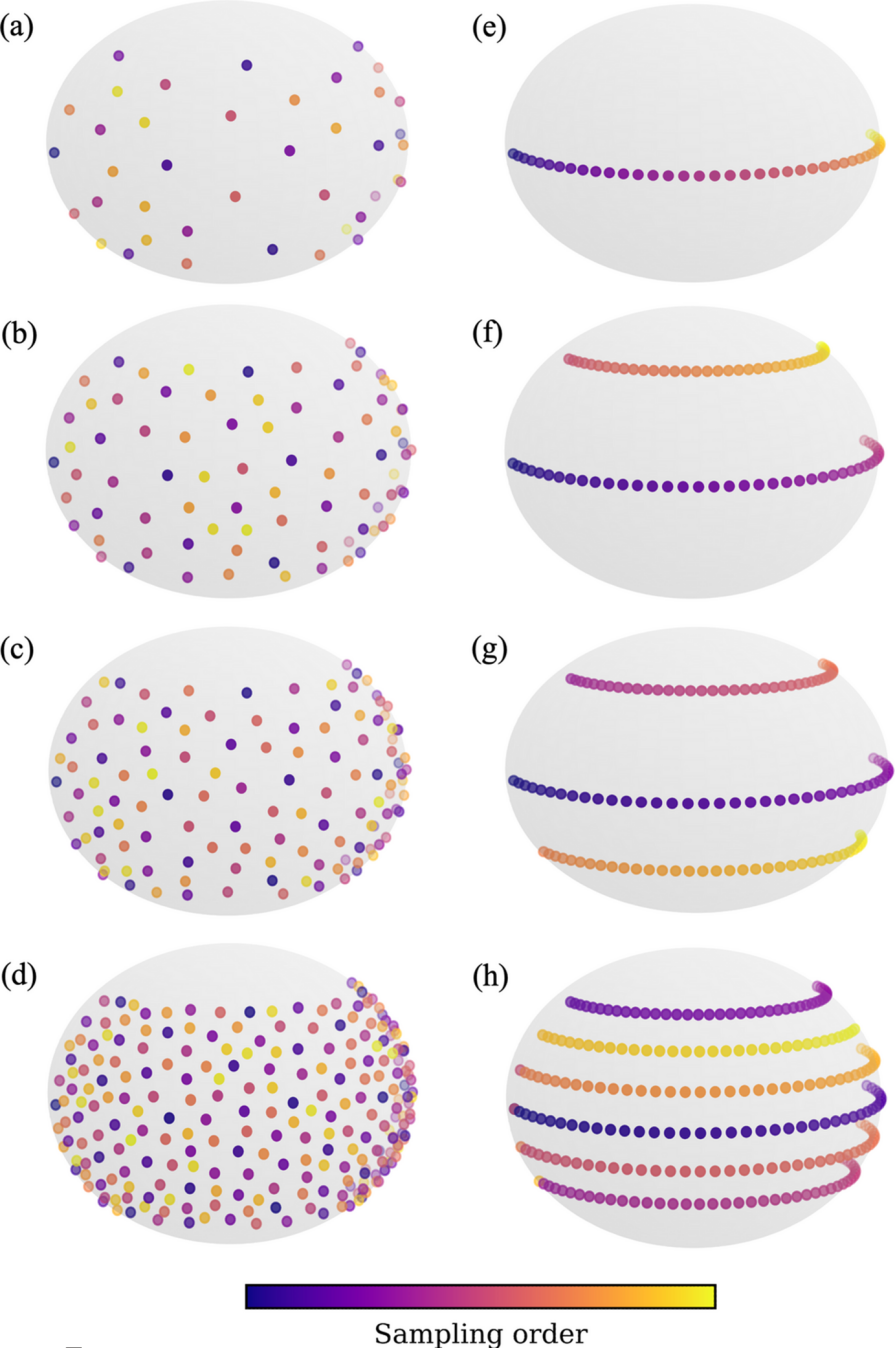
Comparison between random max–min sampling and heuristic experimental acquisition sampling distribution. (*a*)–(*d*) 40, 80, 120 and 240 sampling points generated using the random max–min method. (*e*)–(*h*) The same number of directions taken from the current experimental acquisition. [See Movies S1 and S2 of the supporting information for animation of the 240-point build-up in (*d*) and (*h*) respectively.]

In this simulation, we first generate 240 projection directions using the *random* max–min method. From this set, subsets of 40, 80 and 120 directions are selected to simulate increasingly sparse sampling, as shown in Figs. 7 ▸(*a*) to 7(*d*). For comparison, we use the first 40, 80, 120 and 240 projection directions from the heuristic sampling scheme, as shown in Figs. 7 ▸(*e*) to 7(*h*).

**Figure 8:**
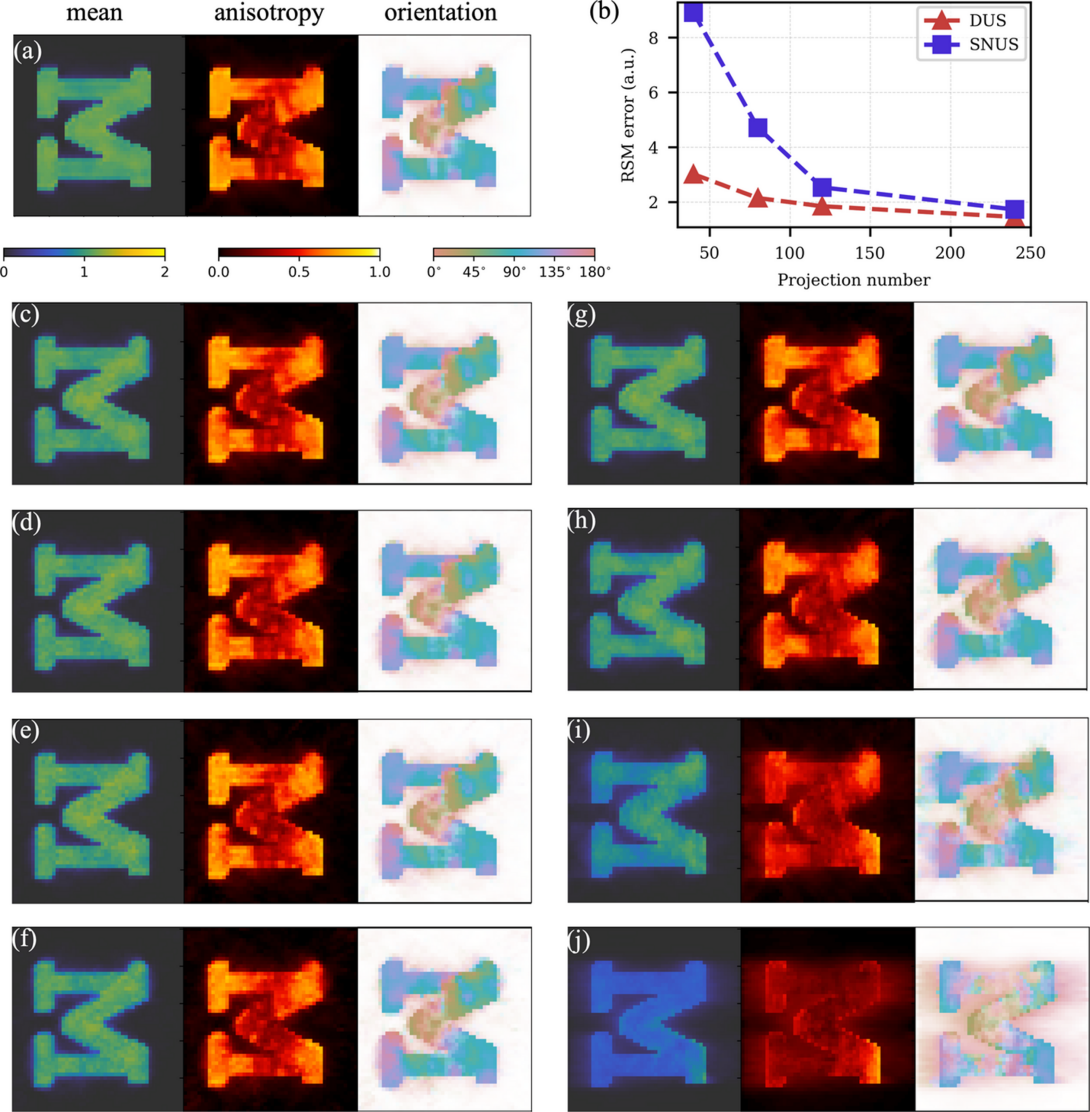
(*a*) Ground truth used as reference, showing the mean intensity, anisotropy level and main orientation within a selected slice. (*b*) RSM error of different projection numbers across different sampling strategies. (*c*)–(*f*) Reconstructions using 240, 120, 80 and 40 sampling points generated by the random max–min method. (*g*)–(*j*) Reconstructions using the same number of sampling points from the heuristic acquisition.

Compared with the ground truth shown in Fig. 8 ▸(*a*), it is evident that the random max–min sampling maintains high reconstruction fidelity even with a reduced number of projections shown in Figs. 8 ▸(*c*) to 8(*f*). The structural features and orientation patterns are largely preserved down to even 40 sampling points. In contrast, the reconstructions based on the heuristic acquisition [Figs. 8 ▸(*g*) to 8(*j*)] degrade more rapidly with fewer projections, and the result with only 80 projections already fails to recover meaningful anisotropy or orientation information, which is consistent with the RSM error curve in Fig. 8 ▸(*b*). This comparison further highlights the advantage of optimized sampling strategies over traditional fixed acquisition sequences.

Overall, both from the experimental and simulation data, our results show that the proposed DUS strategy provides sufficiently uniform angular coverage. It achieves reconstruction quality comparable with ideal SUS, as measured by intensity error, kernel coefficient error, orientation alignment and spatial cross-correlation. Moreover, it supports real-time decision making during acquisition, as the next-few-projection intensity error offers a reliable basis for adaptive sampling. We further note that the strategy is sample-dependent: samples with greater heterogeneity in real or reciprocal space typically require more projections, and no universal criterion can be defined for the optimal number.

## 4. Conclusions and outlook

In this work, a real-time SAXSTT acquisition framework that combines dynamic uniform sampling with online error feedback was introduced to improve efficiency without compromising reconstruction quality. By monitoring the intensity error gradient, the presented framework terminates measurements when additional projections offer minimal improvement. Quantitative results demonstrate up to a sevenfold reduction in acquisition time compared with the heuristic scheme, with further reduction possible for higher error tolerances.

In practical experiments, implementing this framework requires both precise sample rotation and real-time data processing. Modern multi-axis positioning systems can offer the speed and accuracy necessary to support dynamic sampling. For suitable experimental setups, both the additional positioning overhead and the reconstruction time can be small compared with the raster-scan time required for each projection, making real-time feedback feasible during acquisition. Moreover, the reconstruction and error evaluation could be updated after small batches of newly acquired projections rather than after every single projection, reducing the computational overhead while still supporting early termination decisions. Although alignment corrections and error evaluation must adapt to continuously incoming data, this can be achieved with only minor changes to existing SAXSTT pipelines. Together, these factors make the framework adaptable for routine use at synchrotron facilities.

## Supplementary Material

Supplementary movie S1. DOI: 10.1107/S1600577526005308/tv5089sup1.gif

Supplementary movie S2. DOI: 10.1107/S1600577526005308/tv5089sup2.gif
