# Supplementary figures and images for: Dynamic sampling for SAXSTT: towards real-time measurement adaptation
Source: J Synchrotron Radiat. 2026 Jun 19;33(Pt 4):1159–68. doi: 10.1107/S1600577526005308 (PMC13344601; doi:10.1107/S1600577526005308)

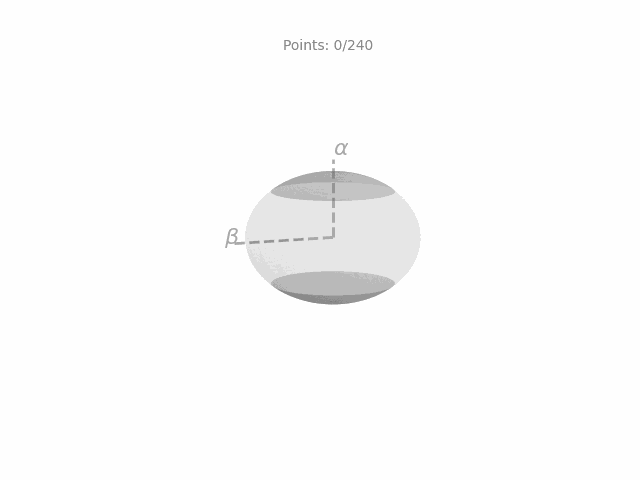

Supplement: Supplementary file 1 [file s-33-01159-sup1.gif]

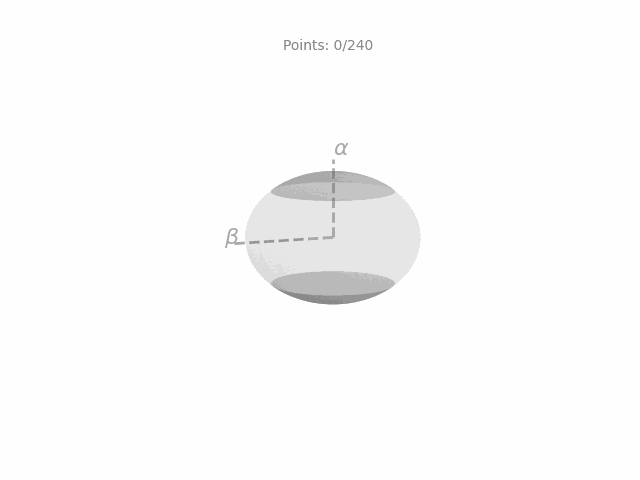

Supplement: Supplementary file 2 [file s-33-01159-sup2.gif]
